# Supplementary figures and images for: Distinct roles of neuronal phenotypes during neurofeedback adaptation
Source: PLoS One. 2026 Jul 10;21(7):e0351053. doi: 10.1371/journal.pone.0351053 (PMC13354095; doi:10.1371/journal.pone.0351053)

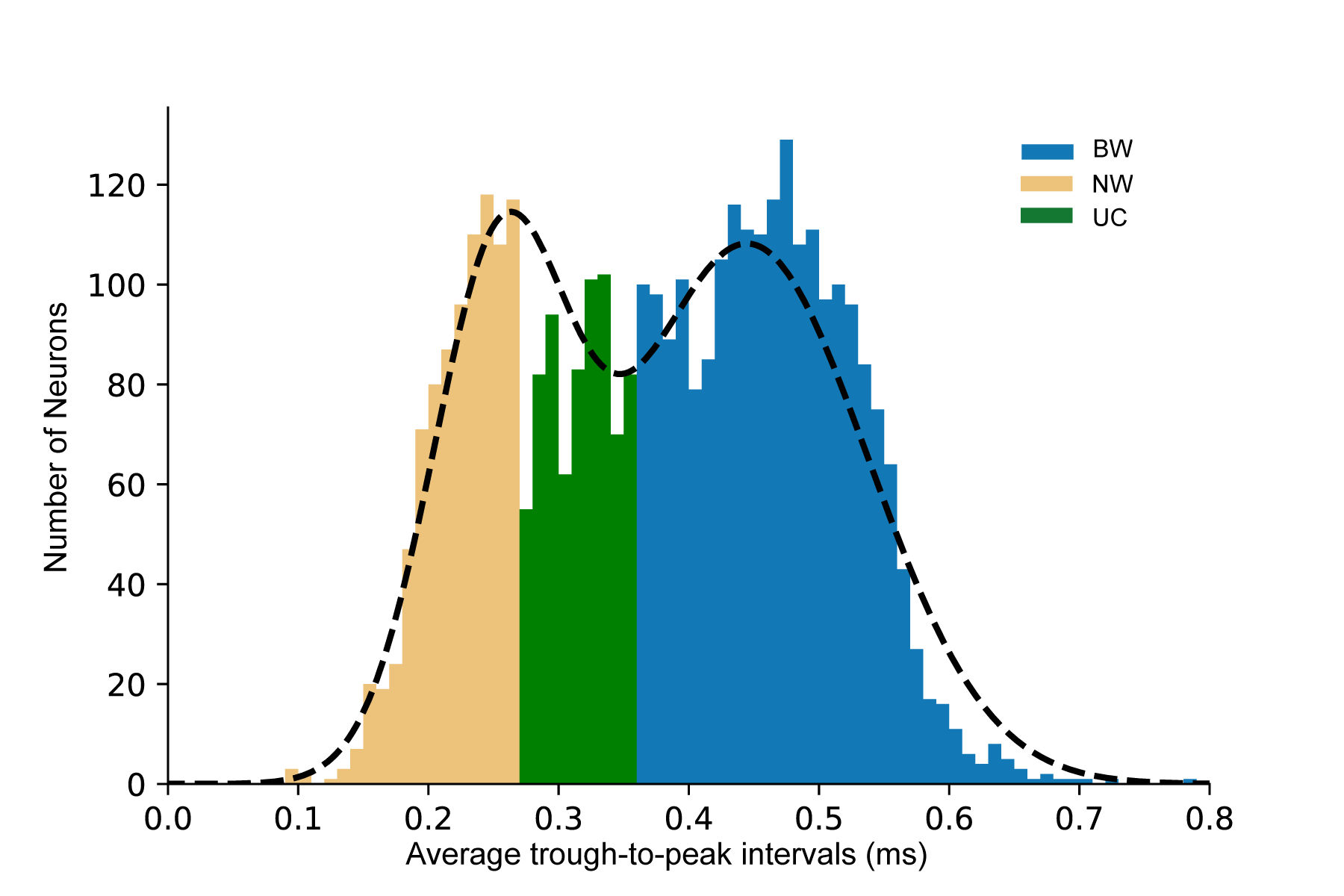

Supplement: S1 Fig — The reductions in the Akaike Information Criterion (Monkey A: −1592 to −1860; Monkey B: −5265 to −5594) and the Bayesian Information Criterion (Monkey A: −1581 to −1835; Monkey B: −5253 to −5563) show that two mixed Gaussian models were better than a single Gaussian model. (TIF) [file pone.0351053.s001.tif]

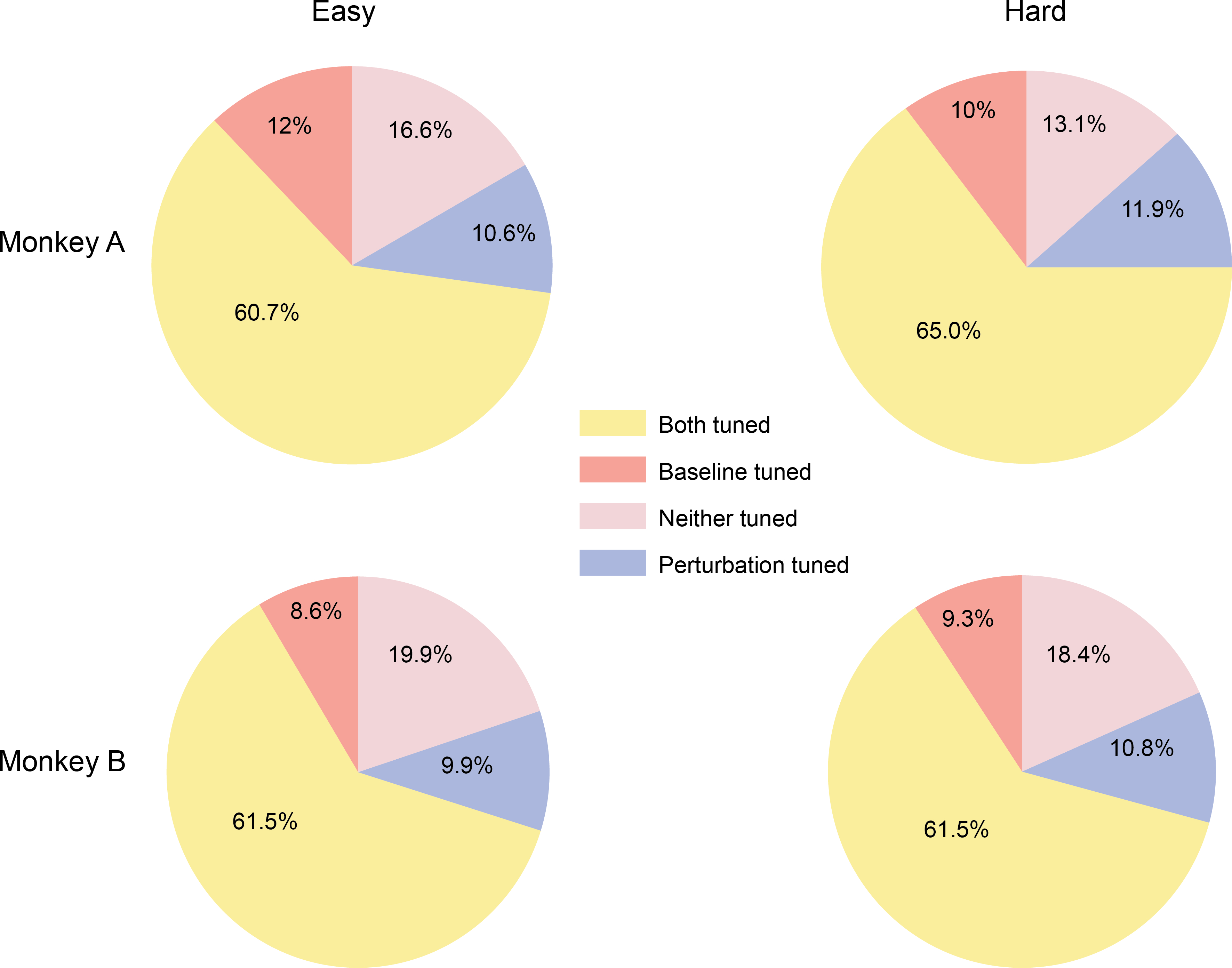

Supplement: S2 Fig — In both task conditions, around 60% of total neurons are tuned in both baseline and perturbation blocks (yellow). Only around 10% of total neurons were only baseline tuned (red) or perturbation tuned (purple). Around 15% of total neurons were not sensitive to directions in both baseline and perturbation. (TIF) [file pone.0351053.s002.tif]

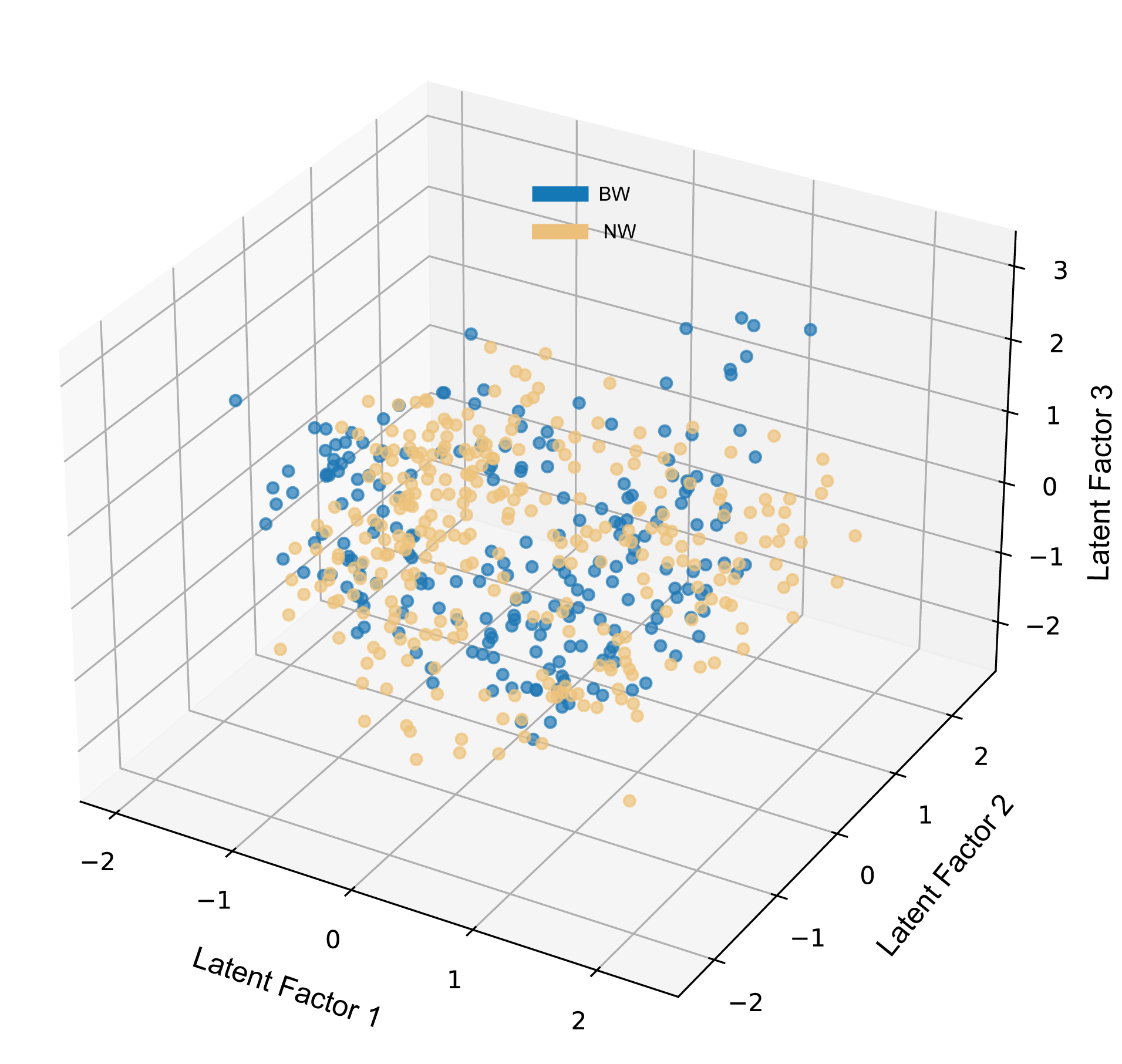

Supplement: S3 Fig — There was no significant difference between the latent repertoire between baseline and perturbation (t2 = 1.75e-29, p = 1, Hotelling’s T^2 test). (TIF) [file pone.0351053.s003.tif]
