# Supplementary material for: Distinct roles of neuronal phenotypes during neurofeedback adaptation
Source: PLoS One. 2026 Jul 10;21(7):e0351053. doi: 10.1371/journal.pone.0351053 (PMC13354095; doi:10.1371/journal.pone.0351053)
Supplement: Attachment — Submitted filename: Response to reviewers.pdf [file pone.0351053.s005.pdf]

Distinct roles of neuronal phenotypes during neurofeedback adaptation

PONE-D-25-29925

We would like to express our sincere gratitude to the editor and the reviewers for their insightful evaluation and constructive feedback on our manuscript. We are encouraged by the recognition of our study as "a valuable and timely contribution to the field" and that our work "addresses an important and often overlooked question: how different cortical cell types contribute to learning adaptation during BMI control". We have taken onboard the reviewers' thoughtful comments regarding the distinction between BMI and natural motor learning, as well as the need for further methodological transparency, and have used this to significantly strengthen the manuscript. We have carefully addressed each concern, which we believe has greatly enhances the clarity and rigor of our findings. This revised version not only resolves the previous discrepancies but also more robustly demonstrates the distinct roles of cortical cell types in neural adaptation, fully aligning with the publication standards of *PLOS One*.

EDITOR:

The paper would benefit from a clearer distinction between BMI learning and natural motor learning, as these processes may involve distinct neural mechanisms that are not fully acknowledged here

We thank the Editor and Reviewer for this thoughtful and important comment. We agree that BMI learning and natural motor learning, while related, are not identical processes and may rely on partially distinct neural mechanisms. To avoid confusion and improve conceptual clarity, we have revised the manuscript to more precisely describe the phenomenon under study as "neurofeedback adaptation" rather than motor learning. In addition, we have added a dedicated paragraph in the Introduction to explicitly differentiate BMI-based adaptation, which relies on an experimenter defined neural-to-cursor mapping and lacks proprioceptive feedback from natural motor learning occurring within an intact sensorimotor loop. These revisions clarify the scope of our findings and appropriately contextualize BMI adaptation as a related but mechanistically distinct learning process.

7. We note that Figure 1 in your submission contain copyrighted images. All PLOS content is published under the Creative Commons Attribution License (CC BY 4.0), which means that the manuscript, images, and Supporting Information files will be freely available online, and any third party is permitted to access, download, copy, distribute, and use these materials in any way, even commercially, with proper attribution. For more information, see our copyright guidelines: http://journals.plos.org/plosone/s/licenses-and-copyright.

> We appreciate the Editor bringing this to our attention. We carefully reviewed all components of Figure 1 and confirmed that the only element that might appear similar to commonly used illustrations: the monkey head outline which was original artwork created specifically for this project by a member of our laboratory (she's very talented!). It was not adapted from or based on any copyrighted source. All other elements of the figure were also generated entirely by the authors. Therefore, Figure 1 does not contain copyrighted material and is fully compatible with the CC BY 4.0 license.

REVIEWER #1:

a. The sentence “On average 37.6 ± 6.2 units in monkey A and 91.6 ± 19.3 units in monkey B were used” does not clarify whether all these units were actively used for BMI control or some were indirect neurons. Was this variability due to recording instability, session-to-session variability, or intentional changes in unit selection? Did it decrease substantially over the sessions? It would be useful to provide a graph of the number of units recorded each day from each NHP.

We thank the reviewer for pointing out the need for clarification. All units reported in the manuscript were actively used as control units in the BMI decoder; no indirect or non-control neurons were included. The variability in the number of recorded units across days reflects natural recording stability fluctuations associated with chronic microelectrode arrays rather than intentional changes in unit selection. As is typical with long-term implants, we observed a gradual downward trend in the total number of isolatable units over months, consistent with expected tissue responses around the array. However, the variation did not reflect substantial day-to-day drops within individual sessions. We have now revised the Methods section to clarify that (1) all units were used for BMI control, (2) variability was driven by chronic recording stability rather than experimental choices, and (3) a decline occurred over the implant duration.

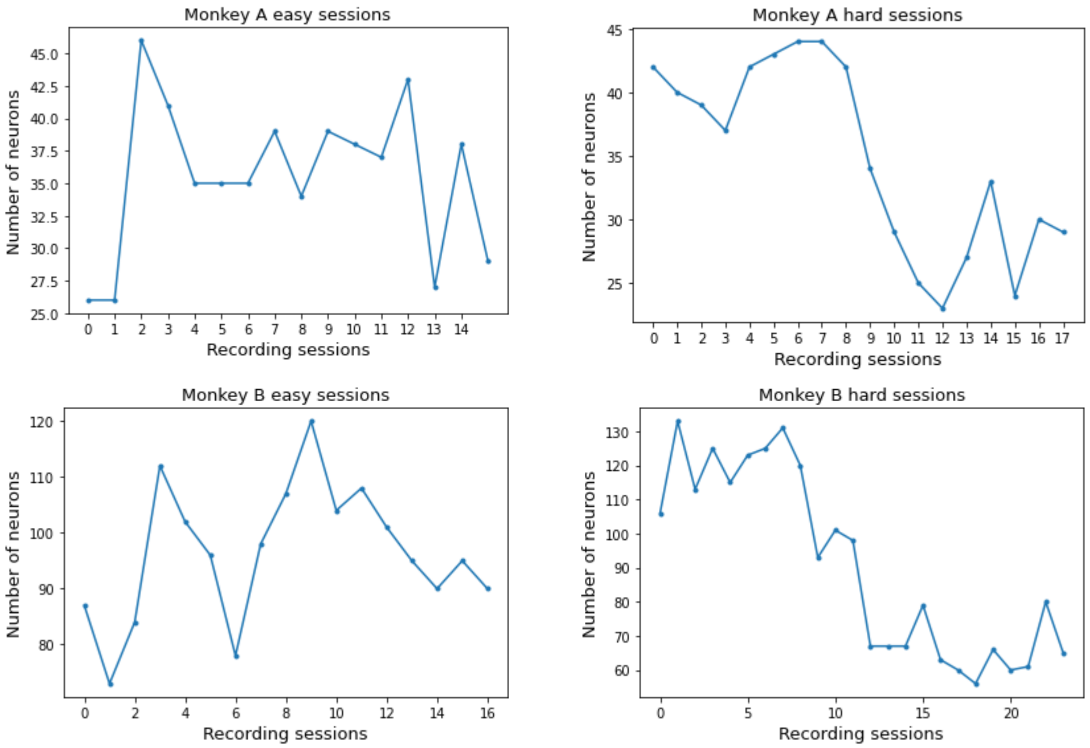

b. If the neurons controlling the BMI change day to day substantially, did it affect performance? Is there a correlation between the number of neurons in the ensemble and learning?

We appreciate the reviewer’s question regarding the relationship between ensemble size and behavioral performance. In our dataset, session-to-session variability in the number of available units did not measurably affect BMI performance. This is expected because

the Kalman filter used in this study requires only a small number of well-modulated units to provide stable two-dimensional velocity estimates; in principle, two directionally informative neurons are sufficient for cursor control. Across sessions, both monkeys demonstrated consistent baseline performance despite natural fluctuations in unit count, indicating that the decoder operated well within a regime where the number of neurons was more than adequate for robust control. Consistent with this, we did not observe a significant correlation between ensemble size and behavioral performance or learning metrics. We have added clarifying text in the manuscript to make this explicit.

c. The description “the subject performs a 10-minute center-out BMI task with gradually decaying computer assistance” is too vague and omits critical methodological details. The paper does not specify the type of assistance provided or how it was implemented. Was this a standard closed-loop decoder adaptation (CLDA) approach, or a different method? Without this information, it is impossible to interpret how the animals transitioned from assisted to independent control. The authors must clearly describe the algorithm or procedure used, including how assistance decayed over time and how this affected cursor dynamics. This information is essential for reproducing the experiment and understanding the learning process.

We thank the reviewer for highlighting the need for a more detailed description of the decoder-assistance procedure. We have now substantially expanded this section of the Methods to clarify that we implemented a standard closed-loop decoder adaptation (CLDA) procedure, following prior work by Orsborn et al. (2014) and Dangi et al. (2014). Specifically, we used a recursive maximum likelihood adaptation rule during a brief 5–10 minute calibration period prior to the main task. During CLDA, the cursor velocity was driven by a weighted combination of the neural decoder output and straight-line computer assistance. The assistance weight decreased linearly from 10% to 0% across the adaptation period, allowing the decoder to converge toward an intuitive mapping while the animal gradually assumed full control. Importantly, no CLDA or computer assistance was applied during the main baseline or perturbation blocks**.** These revisions clarify how the subjects transitioned from assisted to fully independent control and provide sufficient detail for experimental reproduction. The full procedural description is now included in the revised Methods section.

d. The distinction between easy and hard trials is introduced only in the results, leaving key aspects of experimental design unclear. The manuscript lacks definitions of each condition, their sequence, whether the order was randomized, and the total number of trials. Without this information, it is difficult to evaluate the task or interpret the findings. The authors should include a complete task description in the methods.

We thank the reviewer for noting the need for clearer definitions of the easy and hard task conditions. We have now revised the Methods section to explicitly describe the perturbation magnitudes, session structure, and trial counts. Each recording session consisted of one perturbation condition only: either the easy condition (50° rotation) or the hard condition (90° rotation). Sessions were not intermixed; all easy-condition sessions were completed before the hard condition sessions. For Monkey A, we recorded 16 easy and 18 hard sessions; for Monkey B, 17 easy and 24 hard sessions. Within each session, monkeys performed 336 trials in the baseline block and 384 trials in the perturbation block. These details have now been added to the Methods section to ensure clarity and reproducibility.

2- Classifying neuronal populations using only one parameter, spike waveform width, is an accepted but limited method. While this method appears in several NHP studies, rodent research typically uses multiple features (e.g., waveform, firing rate, autocorrelograms) to ensure more reliable separation of inhibitory and excitatory neurons. Although single-metric classification may be standard in some NHP work, this limitation should at least be acknowledged in the discussion, given the central focus on cell-type-specific contributions.

We thank the reviewer for this insightful comment. We agree that classifying neurons based solely on waveform width is a useful but limited approach, and that multispectral clustering using additional features (e.g., firing rate, autocorrelogram structure) is more common in rodent electrophysiology. In nonhuman primate recordings, however, single-metric spike-width classification is widely used and has been shown to reliably separate putative inhibitory and excitatory neurons. Our classification yielded NW proportions (27.2% in Monkey A; 19.3% in Monkey B) that fall well within the expected 15–30% range reported in prior NHP studies. Nevertheless, we have now added explicit acknowledgment of this methodological limitation in the Discussion and clarified that future work incorporating multi-feature classification or optogenetic tagging would further strengthen neuron-type identification. The revised text appears in the Discussion section.

3- Even accepting the use of a single waveform metric, the authors' implementation raises concerns. The data clearly shows a bimodal distribution with reasonable Gaussian fits, yet the chosen cutoff excludes nearly half of the NW distribution while retaining most of the BW population. This asymmetry is difficult to justify. The studies used as references are not consistent with the cutoff, so this choice is neither standard nor well-motivated. Even more since they do seem to use the full Gaussian fit (e.g. Ardid et al. 2015 Fig.1D, Oemisch et al. 2015 Fig. 6C, Oemisch et al. 2019 Fig. 8B-G) Selectively trimming one population risks biasing the analysis and likely biases the role of NW neurons in adaptation. If the authors wish to maintain this threshold, they must justify why this specific cutoff is preferable to other options, such as using the full Gaussian fits, and provide a supplemental analysis showing how the results change with more symmetrical thresholds. This is critical to assess whether the conclusions depend on the current classification scheme.

We thank the reviewer for raising this important point. Our classification procedure follows the same likelihood-ratio approach used in Ceccarelli et al. (2023) and other primate electrophysiology studies. In this method, a two-Gaussian mixture model is fit to the trough-to-peak interval distribution, and neurons are assigned to the NW or BW class only when the probability of belonging to that class is at least 10× higher than the probability of belonging to the other class. This strategy provides a conservative and principled way to classify neurons by retaining only those with high assignment certainty.

The asymmetry noted by the reviewer arises naturally from the different variances of the NW and BW waveform distributions. The NW waveform distribution is considerably narrower, which shifts the 10× likelihood boundary closer to its peak. This does not reflect selective removal of NW neurons but rather the statistical structure of the underlying data, which is the same reason similar asymmetry appears in the published literature using this method. Because our goal was to compare neuron groups with high classification confidence, we chose the likelihood-ratio approach instead of the Gaussian-intersection method, which assigns a larger fraction of borderline neurons whose waveform properties overlap. We have now clarified this rationale in the manuscript and added a brief acknowledgment in the Discussion noting that, while alternative thresholds exist, our conclusions rely on the strongly classified subset of neurons and therefore remain robust to the conservative nature of the cutoff.

4- Several statements in the manuscript conflate BMI learning with natural motor learning without sufficient support or discussion. While it is very likely that BMI adaptation recruits the same mechanisms, potentially by hijacking the same cortico-striatal-thalamo-cortical loops involved in motor learning, there is also clear evidence of differences. Prior work, including Zippi et al. (2023) (https://doi.org/10.1038/s41598-023-44405-y), has shown distinct neural representations during BMI versus manual control across the motor cortex, prefrontal cortex, and striatum. These differences may arise from the number and identity of neurons recruited in each case or because they do use different systems altogether. The authors should acknowledge this nuance, avoid treating BMI learning as equivalent to motor learning, and discuss these distinctions explicitly.

We thank the reviewer for this insightful comment. We agree that BMI-based adaptation and natural motor learning share overlapping computational principles but are not equivalent processes. To prevent confusion, we have revised the manuscript to refer to the phenomenon under study as “neurofeedback adaptation” rather than “motor learning,” except when discussing prior literature. We also added text in the Introduction and Discussion explicitly noting that BMI adaptation occurs within an artificial neural-to-cursor mapping and lacks the proprioceptive and musculoskeletal feedback present in natural movement, consistent with evidence from Zippi et al. (2023) and others showing distinct neural representations between BMI and manual control. These revisions clarify that our findings pertain specifically to neurofeedback adaptation within a BMI framework and are not intended to imply direct equivalence with natural motor learning.

5- Figure 2C shows a clear and important result: NW neurons decrease their firing rate from easy to hard conditions, while BW neurons increase theirs. This finding directly contradicts the overall narrative of the paper, which emphasizes a dominant role for NW neurons in adaptation. Despite this, the authors barely mention the result and provide no interpretation. Is it possible that the increased firing rate in BW neurons reflects a compensatory response to the harder perturbation? If so, how does this fit with the conclusion that NW neurons play the dominant role in adaptation? This apparent contradiction needs to be addressed directly.

We thank the reviewer for raising this important point. We agree that the firing-rate differences observed between the easy and hard conditions require clarification. Because our recordings were obtained across different sessions, the neurons contributing to the easy-condition dataset are not the same neurons contributing to the hard-condition dataset. Therefore, cross-session comparisons of mean firing rate cannot be interpreted as learning-related increases or decreases in activity. These differences instead reflect normal variability in the set of isolatable units recorded each day.

Our intention in presenting Figure 2C was not to use firing-rate changes across conditions to infer adaptation-related effects, but rather to illustrate the intrinsic firing-rate differences between waveform classes. Based on waveform shape, NW neurons are more likely to correspond to interneurons, whereas BW neurons are more likely to correspond to pyramidal cells, and the observed firing-rate pattern is consistent with these general physiological trends reported in the literature. This interpretation relates to intrinsic cell-type properties rather than task-dependent modulation.

Crucially, all conclusions about the dominant role of NW neurons in adaptation are based on within-session comparisons, such as unit contribution, pSOT coordination, and ω^2 encoding, where the same neurons are compared across baseline and perturbation blocks. These within-session metrics directly capture how each neuronal phenotype responds to the perturbation and therefore form the proper basis for our interpretation. We have revised the Results and Discussion to clarify this distinction.

6- Is there a difference in ω2 between early and late adaptation, or between baseline and late adaptation? How much does the explained variance change between easy and hard conditions?

We appreciate the reviewer's question regarding temporal structure in the ω^2 analysis. Our ω^2 measure was computed using the entire baseline block and the entire perturbation block, rather than subdividing these blocks into early and late phases. This design choice reflects the statistical properties of ω^2: reliable estimation requires a sufficiently large number of trials per target direction, and dividing blocks into smaller segments would substantially reduce the number of observations, leading to unstable estimates that cannot be meaningfully interpreted. For this reason, early–late comparisons are not feasible within the framework of our ω^2 analysis.

Regarding the reviewer's second question, we did evaluate how explained variance differed between easy and hard perturbation conditions. As reported in the Results, both NW and BW neurons exhibited significantly higher ω^2 values in the hard condition than in the easy condition for both monkeys ($p < 0.001$, two-sample t-test). Thus, while ω^2 cannot be partitioned into early and late phases, our analysis already captures how task difficulty modulates the strength of neuronal encoding during adaptation. We have added a brief clarification in the Methods to make this explicit.

7- Data is not available in a public repository and the Github address that was given is not a valid address.

8- References:

a. Ref 19 has no authors

b. The DOI link for ref 69 is incorrect

c. Missing citation for sentence in line 41-43 "the brain adapts quicker to smaller perturbations."

All these concerns have been addressed in the manuscript.

9- Clarity:

a. The statement in line 279-281 "However, the proportion of well-tuned neurons in the NW and BW neuron 280 subpopulations were significantly different in both subjects (Fig. 2E)." is confusing as it implies that there is a difference between the two subjects (while the figure clearly states that in both subject the difference between the groups is the same).

b. Line 372-374 "Since FA did not identify differences in neural activity between easy and hard task conditions in Monkey A, we applied ω2 to compare single-neuron responses during trials across neuron types." seems to imply that ω2 is analyzed only in monkey A.

c. In figure 6A-B does the x-axis represent peak activity or peak ω2? The description says peak ω2, but the label says peak activity.

d. Figure 4 lacks a clear separation or legend that distinguishes monkey A from monkey B.

All these concerns have been addressed in the manuscript.
