# Supplementary material for: Distinct roles of neuronal phenotypes during neurofeedback adaptation
Source: PLoS One. 2026 Jul 10;21(7):e0351053. doi: 10.1371/journal.pone.0351053 (PMC13354095; doi:10.1371/journal.pone.0351053)
Supplement: Attachment — Submitted filename: Response_to_Reviewers_auresp_2.pdf [file pone.0351053.s006.pdf]

Distinct roles of neuronal phenotypes during neurofeedback adaptation

PONE-D-25-29925R1

We would like to thank the reviewer for their thoughtful and constructive comments regarding the neuron classification. We agree with their constructive criticism and have fully addressed the concerns by re-implementing all analyses using the suggested methodology for setting cutoff thresholds. This comprehensive re-evaluation has confirmed that our original results are robust, as the core findings remain preserved under the new criteria. Consequently, we have updated the manuscript and nearly all of the figures and statistical results to reflect the revised methods and corresponding analysis. A detailed account of these changes and the stability of our conclusions follows below.

REVIEWER #1:

1- Figure 2A. Unfortunately, I don't believe the authors adequately addressed previous concerns about the cutoff in the neuron selection. I understand that previous studies have used a 1:10 likelihood criterion as a conservative way to define high-confidence classes (Ceccarelli et al. (2023)). However, in the present dataset (with one Gaussian centered at ~0.25 and the other at ~0.47), the resulting cutoffs appear shifted (0.25 and 0.35) and split (at 0.25) of one of the Gaussian distributions in the middle rather than lying in the overlap region between the two fitted populations (as happens in Ceccarelli et al. (2023). This selection may reduce statistical power and bias the analysis. Moreover, many neurons are excluded and then the analysis may no longer represent the underlying population and may be very dependent on the chosen cutoff.
Is it possible that the histogram is not showing the entire dataset? (so the Gaussian obtained is not centered at 0.25?). Is it possible that the histogram shows the mean trough-to-peak interval while the Gaussian was fit to the raw data? The authors should consider showing the Gaussian fits overlaid on the histogram to assess any misalignment. However, if the Gaussian was fit correctly and the cutoff lies within one Gaussian, the authors should show how the cutoff changes under less stringent likelihood ratios (e.g., 1:5 or 1:3) and confirm that the main result remain unchanged across at least one of these thresholds.

We appreciate the reviewer's careful attention to detail and we have taken the suggestion onboard. Firstly, we would like to confirm that the entire dataset is shown in the histogram in Fig. 2A. Secondly, following the suggestion to assess the stability of our findings under less stringent likelihood ratios, we have re-analyzed the entire dataset using a 1:5 likelihood ratio. As expected, this shifted the NW and BW cutoffs further in the right and left tails of the distributions, respectively.

	NW Threshold: New (Old)	BW Threshold: New (Old)
Monkey A	0.275 ms (0.243 ms)	0.348 ms (0.360 ms)
Monkey B	0.272 ms (0.240 ms)	0.360 ms (0.373 ms)

Regarding the impact of the threshold shift, we found that by relaxing the threshold, we significantly increased the number of neurons included in the analysis, thereby addressing the concern regarding the potential exclusion of representative population data.

	# NW Neurons: New (Old)	# BW: New (Old)	Total # Neurons Recorded
Monkey A	430 (325)	537 (513)	1195
Monkey B	933 (737)	2107 (2146*)	3814

**We would like to note that we previously noted that 2,146 neurons were labeled as BW; however, the correct count under the 1:10 ratio was 2,046. By adopting the 1:5 likelihood ratio and adjusting the BW threshold for Monkey B (from 0.373 ms to 0.360 ms), the number of BW neurons did increase.*

With the neuron classification performed this way, we found that the primary conclusions of the study remained robust under the 1:5 criterion. All previously reported effects were preserved using the 1:5 ratio, with one exception. We updated Figures 2, 3, 4, 5, and 6, and Supplementary Figures 1 and 3, to reflect visualization of the re-analyzed data. The corresponding statistical analyses were also updated accordingly and incorporated into the revised manuscript. The singular result for which statistical significance was not preserved was the main effect of neuron type on the peak percentage of explained variance (peak ω^2; see Figure 6) in one of the subjects. Specifically, two-way ANOVA with peak ω^2 as the dependent variable found that the main effect of neuron type had a p-value of p = 0.052 for monkey A (the main effect is significant for monkey b is still significant: p << 0.001), which is slightly above the conventional significance level of 0.05. We note in the revised text that the effect is trending for monkey A and significant for monkey B.

2- Figure 2C. I understand that firing-rate differences between these conditions cannot be interpreted as within-neuron learning effects. However, Figure 2C still shows a clear population-level pattern. NW neurons have lower firing rates in the hard condition than in the easy condition. The converse is true for BW neurons. Even if this reflects differences in the neurons recorded across sessions, the pattern remains in the data and should be addressed. The cross-session design also does not prevent statistical comparison of the populations. A simple independent-samples test could resolve this concern. If the effect were significant, it could indicate a shift in the types of neurons engaged across different task conditions.

We agree that we should have set up our original analysis to capture this in the way that you suggest. To better comprehensively reveal what effects are truly present in the data depicted in Fig 2C, we have performed a two-way ANOVA that simultaneously tests for main effects between task conditions and neuron types, as suggested.

**TWO-WAY ANOVA**	**p-value (Monkey A)**	**p-value (Monkey B)**
Main effect: task condition	p = 0.637	p = 0.773
Main effect: neuron type	p = 0.000006	p = 8.15 × 10⁻63
Interaction	p = 0.0026	p = 0.348

Our results confirm a significant effect of neuron type, as originally reported in the paper, and this result holds for both subjects (see summary table below for your convenience). While the data presented in Fig 2C does visually suggest there is may be a main effect of task condition, we found that task condition was not a significant main effect and that this also held true for both subjects. Although a significant interaction was detected in one subject (monkey A), the absence of a main effect of task condition and the lack of a reproducible interaction across both subjects suggests that these trends likely reflect session-specific variance rather than a fundamental shift in population engagement. We have added these statistical details to the manuscript to clarify that while neuron type is a robust predictor of firing rate, task difficulty did not exert a consistent or significant influence on firing rates.

We have updated the following text in the Neuron Classification subsection within the Results:

We evaluated how firing rate activity of neurons differ under this classification and across task conditions. The absence of an effect of task condition (Monkey A: p = 0.637; Monkey B: p = 0.773; main effect of neuron type, two-way ANOVA) and the lack of a reproducible interaction between task condition and neuron type across both subjects (Monkey A: p = 0.0026; Monkey B: p = 0.348; interaction effect, two-way ANOVA) suggest that the primary driver of differences in firing rate is the neuron type (Monkey A: p <0.001; Monkey B: p<0.001; main effect of neuron type, two-way ANOVA). The mean firing rate of NW neurons during cursor movements was significantly higher than that of BW neurons (Fig. 2C) under both easy and hard task conditions. This high firing rate of NW neurons, which is typical of inhibitory neuron, is also consistent with findings from previous studies[49,58,60].

(minor) Data was still not accessible through the github link provided by the authors.

We recently changed how our data is hosted so that it is no longer linked to individual trainee Github accounts. Access to our data is restricted now while under review, but if accepted the data will be freely available on figshare (this platform allows for larger repositories). We will comply with the journal's requirements to make the data available upon publication but understand from their policy that it is permissible to maintain the data privately during the manuscript review process.
