# Supplementary material for: Distinct roles of neuronal phenotypes during neurofeedback adaptation
Source: PLoS One. 2026 Jul 10;21(7):e0351053. doi: 10.1371/journal.pone.0351053 (PMC13354095; doi:10.1371/journal.pone.0351053)
Supplement: Peer review history

# Author comment: Author response to Decision Letter 0

**DOI:** 10.1371/journal.pone.0351053.r001

**Transfer Alert**

This paper was transferred from another journal. As a result, its full editorial history (including decision letters, peer reviews and author responses) may not be present.

2 Jun 2025

# Peer review: Decision Letter 0

**DOI:** 10.1371/journal.pone.0351053.r002

**Authors:** Marie-Constance Corsi

12 Nov 2025

PONE-D-25-29925

Distinct roles of neuronal phenotypes during neurofeedback adaptation

PLOS ONE

Dear Dr. Santacruz,

Thank you for submitting your manuscript to PLOS ONE.

Your study presents a timely and important investigation into the cell-type-specific contributions to learning adaptation during brain-machine interface (BMI) control, addressing a critical but under-explored area of research. The study is well-executed and provides compelling evidence for the distinct roles of different cortical cell types in BMI learning.

However, the paper would benefit from a clearer distinction between BMI learning and natural motor learning, as these processes may involve distinct neural mechanisms that are not fully acknowledged here. Additionally, several methodological aspects require further clarification, including the criteria for neuron selection, the specifics of computer assistance during tasks, and the operational definitions of experimental conditions (e.g., "easy" vs. "hard" trials). The reliance on spike waveform width alone for neuron classification also raises concerns about potential bias, particularly given the lack of justification for the chosen cutoff.

Finally, the manuscript includes findings that appear to contradict its central arguments, yet these discrepancies are not sufficiently addressed. To strengthen the study’s conclusions, additional analyses, such as the relationship between neuron ensemble size and performance, changes in explained variance across conditions, and the availability of data for public access, would be essential to ensure both the robustness and reproducibility of the results.

Therefore, after careful consideration, we feel that it has merit but does not fully meet PLOS ONE’s publication criteria as it currently stands. Therefore, we invite you to submit a revised version of the manuscript that addresses the points raised during the review process.

Please submit your revised manuscript by Dec 27 2025 11:59PM. If you will need more time than this to complete your revisions, please reply to this message or contact the journal office at plosone@plos.org. When you're ready to submit your revision, log on to https://www.editorialmanager.com/pone/ and select the 'Submissions Needing Revision' folder to locate your manuscript file.

Please include the following items when submitting your revised manuscript:

- A rebuttal letter that responds to each point raised by the academic editor and reviewer(s). You should upload this letter as a separate file labeled 'Response to Reviewers'.
- A marked-up copy of your manuscript that highlights changes made to the original version. You should upload this as a separate file labeled 'Revised Manuscript with Track Changes'.
- An unmarked version of your revised paper without tracked changes. You should upload this as a separate file labeled 'Manuscript'.

If you would like to make changes to your financial disclosure, please include your updated statement in your cover letter. Guidelines for resubmitting your figure files are available below the reviewer comments at the end of this letter.

If applicable, we recommend that you deposit your laboratory protocols in protocols.io to enhance the reproducibility of your results. Protocols.io assigns your protocol its own identifier (DOI) so that it can be cited independently in the future. For instructions see: https://journals.plos.org/plosone/s/submission-guidelines#loc-laboratory-protocols. Additionally, PLOS ONE offers an option for publishing peer-reviewed Lab Protocol articles, which describe protocols hosted on protocols.io. Read more information on sharing protocols at https://plos.org/protocols?utm_medium=editorial-email&utm_source=authorletters&utm_campaign=protocols.

We look forward to receiving your revised manuscript.

Kind regards,

Marie-Constance Corsi

Academic Editor

PLOS ONE

Journal Requirements:

When submitting your revision, we need you to address these additional requirements.

1. Please ensure that your manuscript meets PLOS ONE's style requirements, including those for file naming. The PLOS ONE style templates can be found at https://journals.plos.org/plosone/s/file?id=wjVg/PLOSOne_formatting_sample_main_body.pdf and https://journals.plos.org/plosone/s/file?id=ba62/PLOSOne_formatting_sample_title_authors_affiliations.pdf

2. To comply with PLOS One submissions requirements, in your Methods section, please provide additional information regarding the experiments involving animals and ensure you have included details on methods of anesthesia and/or analgesia, and efforts to alleviate suffering.

3. When completing the data availability statement of the submission form, you indicated that you will make your data available on acceptance. We strongly recommend all authors decide on a data sharing plan before acceptance, as the process can be lengthy and hold up publication timelines. Please note that, though access restrictions are acceptable now, your entire data will need to be made freely accessible if your manuscript is accepted for publication. This policy applies to all data except where public deposition would breach compliance with the protocol approved by your research ethics board. If you are unable to adhere to our open data policy, please kindly revise your statement to explain your reasoning and we will seek the editor's input on an exemption. Please be assured that, once you have provided your new statement, the assessment of your exemption will not hold up the peer review process.

4. We note that the grant information you provided in the ‘Funding Information’ and ‘Financial Disclosure’ sections do not match.

When you resubmit, please ensure that you provide the correct grant numbers for the awards you received for your study in the ‘Funding Information’ section.

5. Please amend your authorship list in your manuscript file to include author Hannah Marie Stealey, Philippe N. Tobler.

6. Please amend the manuscript submission data (via Edit Submission) to include author Hannah M. Stealey, Philippe Tobler.

7. We note that Figure 1 in your submission contain copyrighted images. All PLOS content is published under the Creative Commons Attribution License (CC BY 4.0), which means that the manuscript, images, and Supporting Information files will be freely available online, and any third party is permitted to access, download, copy, distribute, and use these materials in any way, even commercially, with proper attribution. For more information, see our copyright guidelines: http://journals.plos.org/plosone/s/licenses-and-copyright.

We require you to either (1) present written permission from the copyright holder to publish these figures specifically under the CC BY 4.0 license, or (2) remove the figures from your submission:

a. You may seek permission from the original copyright holder of Figure 1 to publish the content specifically under the CC BY 4.0 license.

We recommend that you contact the original copyright holder with the Content Permission Form (http://journals.plos.org/plosone/s/file?id=7c09/content-permission-form.pdf) and the following text:

“I request permission for the open-access journal PLOS ONE to publish XXX under the Creative Commons Attribution License (CCAL) CC BY 4.0 (http://creativecommons.org/licenses/by/4.0/). Please be aware that this license allows unrestricted use and distribution, even commercially, by third parties. Please reply and provide explicit written permission to publish XXX under a CC BY license and complete the attached form.”

Please upload the completed Content Permission Form or other proof of granted permissions as an "Other" file with your submission.

In the figure caption of the copyrighted figure, please include the following text: “Reprinted from [ref] under a CC BY license, with permission from [name of publisher], original copyright [original copyright year].”

b. If you are unable to obtain permission from the original copyright holder to publish these figures under the CC BY 4.0 license or if the copyright holder’s requirements are incompatible with the CC BY 4.0 license, please either i) remove the figure or ii) supply a replacement figure that complies with the CC BY 4.0 license. Please check copyright information on all replacement figures and update the figure caption with source information. If applicable, please specify in the figure caption text when a figure is similar but not identical to the original image and is therefore for illustrative purposes only.

8. If the reviewer comments include a recommendation to cite specific previously published works, please review and evaluate these publications to determine whether they are relevant and should be cited. There is no requirement to cite these works unless the editor has indicated otherwise.

[Note: HTML markup is below. Please do not edit.]

Reviewers' comments:

Reviewer's Responses to Questions

**Comments to the Author**

1. Is the manuscript technically sound, and do the data support the conclusions?

The manuscript must describe a technically sound piece of scientific research with data that supports the conclusions. Experiments must have been conducted rigorously, with appropriate controls, replication, and sample sizes. The conclusions must be drawn appropriately based on the data presented.

Reviewer #1: Yes

**********

2. Has the statistical analysis been performed appropriately and rigorously?

Reviewer #1: Yes

**********

3. Have the authors made all data underlying the findings in their manuscript fully available?

The PLOS Data policy requires authors to make all data underlying the findings described in their manuscript fully available without restriction, with rare exception (please refer to the Data Availability Statement in the manuscript PDF file). The data should be provided as part of the manuscript or its supporting information, or deposited to a public repository. For example, in addition to summary statistics, the data points behind means, medians and variance measures should be available. If there are restrictions on publicly sharing data—e.g. participant privacy or use of data from a third party—those must be specified.

Reviewer #1: No

**********

4. Is the manuscript presented in an intelligible fashion and written in standard English?

PLOS ONE does not copyedit accepted manuscripts, so the language in submitted articles must be clear, correct, and unambiguous. Any typographical or grammatical errors should be corrected at revision, so please note any specific errors here.

Reviewer #1: Yes

**********

5. Review Comments to the Author

Please use the space provided to explain your answers to the questions above. You may also include additional comments for the author, including concerns about dual publication, research ethics, or publication ethics. (Please upload your review as an attachment if it exceeds 20,000 characters)

Reviewer #1: This paper by Zhao Y, Stealey HM, Lu HY, Contreras-Hernandez E, Chang YJ, Tobler PN, and Santacruz SR investigates how different cortical neuron types contribute to learning adaptation when a perturbation is introduced in a brain-machine interface (BMI) task. The authors recorded neural activity from the dorsal premotor cortex of two macaque monkeys using chronically implanted microelectrode arrays and classified neurons into narrow waveform (NW, putative inhibitory interneurons) and broad waveform (BW, putative excitatory pyramidal cells). Their findings show how NW neurons contributed more to the decoder output and played a larger role in learning to overcome the perturbation, suggesting that inhibitory interneurons are key drivers of adaptive control in this context.

We consider this paper a valuable and timely contribution to the field. The study addresses an important and often overlooked question: how different cortical cell types contribute to learning adaptation during BMI control. The experiments are carefully conducted, and the study addresses an important question about cell-type-specific contributions to learning adaptation in BMI tasks. However, the paper leaves several conceptual and methodological points insufficiently addressed. In particular, the authors treat BMI learning and motor learning as equivalent, but these processes may differ in important ways that are not acknowledged. Additionally, some of the key claims regarding neuron classification, decoder contribution, and adaptation dynamics require further clarification or additional data to be fully convincing.

We believe the manuscript could be significantly strengthened by addressing these issues. Below, we outline our major and minor concerns to help the authors refine and clarify the work.

Major concerns:

1- Several crucial details are missing from the methods section.

a. The sentence “On average 37.6 ± 6.2 units in monkey A and 91.6 ± 19.3 units in monkey B were used” does not clarify whether all these units were actively used for BMI control or some were indirect neurons. Was this variability due to recording instability, session-to-session variability, or intentional changes in unit selection? Did it decrease substantially over the sessions? It would be useful to provide a graph of the number of units recorded each day from each NHP.

b. If the neurons controlling the BMI change day to day substantially, did it affect performance? Is there a correlation between the number of neurons in the ensemble and learning?

c. The description “the subject performs a 10-minute center-out BMI task with gradually decaying computer assistance” is too vague and omits critical methodological details. The paper does not specify the type of assistance provided or how it was implemented. Was this a standard closed-loop decoder adaptation (CLDA) approach, or a different method? Without this information, it is impossible to interpret how the animals transitioned from assisted to independent control. The authors must clearly describe the algorithm or procedure used, including how assistance decayed over time and how this affected cursor dynamics. This information is essential for reproducing the experiment and understanding the learning process.

d. The distinction between easy and hard trials is introduced only in the results, leaving key aspects of experimental design unclear. The manuscript lacks definitions of each condition, their sequence, whether the order was randomized, and the total number of trials. Without this information, it is difficult to evaluate the task or interpret the findings. The authors should include a complete task description in the methods.

2- Classifying neuronal populations using only one parameter, spike waveform width, is an accepted but limited method. While this method appears in several NHP studies, rodent research typically uses multiple features (e.g., waveform, firing rate, autocorrelograms) to ensure more reliable separation of inhibitory and excitatory neurons. Although single-metric classification may be standard in some NHP work, this limitation should at least be acknowledged in the discussion, given the central focus on cell-type-specific contributions.

3- Even accepting the use of a single waveform metric, the authors’ implementation raises concerns. The data clearly shows a bimodal distribution with reasonable Gaussian fits, yet the chosen cutoff excludes nearly half of the NW distribution while retaining most of the BW population. This asymmetry is difficult to justify. The studies used as references are not consistent with the cutoff, so this choice is neither standard nor well-motivated. Even more since they do seem to use the full Gaussian fit (e.g. Ardid et al. 2015 Fig.1D, Oemisch et al. 2015 Fig. 6C, Oemisch et al. 2019 Fig. 8B-G)

Selectively trimming one population risks biasing the analysis and likely biases the role of NW neurons in adaptation. If the authors wish to maintain this threshold, they must justify why this specific cutoff is preferable to other options, such as using the full Gaussian fits, and provide a supplemental analysis showing how the results change with more symmetrical thresholds. This is critical to assess whether the conclusions depend on the current classification scheme.

4- Several statements in the manuscript conflate BMI learning with natural motor learning without sufficient support or discussion. While it is very likely that BMI adaptation recruits the same mechanisms, potentially by hijacking the same cortico-striatal-thalamo-cortical loops involved in motor learning, there is also clear evidence of differences. Prior work, including Zippi et al. (2023) (https://doi.org/10.1038/s41598-023-44405-y), has shown distinct neural representations during BMI versus manual control across the motor cortex, prefrontal cortex, and striatum. These differences may arise from the number and identity of neurons recruited in each case or because they do use different systems altogether. The authors should acknowledge this nuance, avoid treating BMI learning as equivalent to motor learning, and discuss these distinctions explicitly.

5- Figure 2C shows a clear and important result: NW neurons decrease their firing rate from easy to hard conditions, while BW neurons increase theirs. This finding directly contradicts the overall narrative of the paper, which emphasizes a dominant role for NW neurons in adaptation. Despite this, the authors barely mention the result and provide no interpretation. Is it possible that the increased firing rate in BW neurons reflects a compensatory response to the harder perturbation? If so, how does this fit with the conclusion that NW neurons play the dominant role in adaptation? This apparent contradiction needs to be addressed directly.

6- Is there a difference in ω2 between early and late adaptation, or between baseline and late adaptation? How much does the explained variance change between easy and hard conditions?

7- Data is not available in a public repository and the Github address that was given is not a valid address.

Minor concerns:

8- References:

a. Ref 19 has no authors

b. The DOI link for ref 69 is incorrect

c. Missing citation for sentence in line 41-43 “the brain adapts quicker to smaller perturbations.”

9- Clarity:

a. The statement in line 279-281 “However, the proportion of well-tuned neurons in the NW and BW neuron 280 subpopulations were significantly different in both subjects (Fig. 2E).” is confusing as it implies that there is a difference between the two subjects (while the figure clearly states that in both subject the difference between the groups is the same).

b. Line 372-374 “Since FA did not identify differences in neural activity between easy and hard task conditions in Monkey A, we applied ω2 to compare single-neuron responses during trials across neuron types.” seems to imply that ω2 is analyzed only in monkey A.

c. In figure 6A-B does the x-axis represent peak activity or peak ω2? The description says peak ω2, but the label says peak activity.

d. Figure 4 lacks a clear separation or legend that distinguishes monkey A from monkey B.

**********

6. PLOS authors have the option to publish the peer review history of their article (what does this mean?). If published, this will include your full peer review and any attached files.

If you choose “no”, your identity will remain anonymous but your review may still be made public.

**Do you want your identity to be public for this peer review?**  For information about this choice, including consent withdrawal, please see our Privacy Policy.

Reviewer #1: **Yes:**  Nuria Vendrell-Llopis

**********

[NOTE: If reviewer comments were submitted as an attachment file, they will be attached to this email and accessible via the submission site. Please log into your account, locate the manuscript record, and check for the action link "View Attachments". If this link does not appear, there are no attachment files.]

To ensure your figures meet our technical requirements, please review our figure guidelines: https://journals.plos.org/plosone/s/figures

You may also use PLOS’s free figure tool, NAAS, to help you prepare publication quality figures: https://journals.plos.org/plosone/s/figures#loc-tools-for-figure-preparation.

NAAS will assess whether your figures meet our technical requirements by comparing each figure against our figure specifications.

# Author comment: Author response to Decision Letter 1

**DOI:** 10.1371/journal.pone.0351053.r003

11 Feb 2026

EDITOR:

The paper would benefit from a clearer distinction between BMI learning and natural motor learning, as these processes may involve distinct neural mechanisms that are not fully acknowledged here

We thank the Editor and Reviewer for this thoughtful and important comment. We agree that BMI learning and natural motor learning, while related, are not identical processes and may rely on partially distinct neural mechanisms. To avoid confusion and improve conceptual clarity, we have revised the manuscript to more precisely describe the phenomenon under study as “neurofeedback adaptation” rather than motor learning. In addition, we have added a dedicated paragraph in the Introduction to explicitly differentiate BMI-based adaptation, which relies on an experimenter defined neural-to-cursor mapping and lacks proprioceptive feedback from natural motor learning occurring within an intact sensorimotor loop. These revisions clarify the scope of our findings and appropriately contextualize BMI adaptation as a related but mechanistically distinct learning process.

7. We note that Figure 1 in your submission contain copyrighted images. All PLOS content is published under the Creative Commons Attribution License (CC BY 4.0), which means that the manuscript, images, and Supporting Information files will be freely available online, and any third party is permitted to access, download, copy, distribute, and use these materials in any way, even commercially, with proper attribution. For more information, see our copyright guidelines: http://journals.plos.org/plosone/s/licenses-and-copyright.

We appreciate the Editor bringing this to our attention. We carefully reviewed all components of Figure 1 and confirmed that the only element that might appear similar to commonly used illustrations: the monkey head outline which was original artwork created specifically for this project by a member of our laboratory (she’s very talented!). It was not adapted from or based on any copyrighted source. All other elements of the figure were also generated entirely by the authors. Therefore, Figure 1 does not contain copyrighted material and is fully compatible with the CC BY 4.0 license.

REVIEWER #1:

The sentence “On average 37.6 ± 6.2 units in monkey A and 91.6 ± 19.3 units in monkey B were used” does not clarify whether all these units were actively used for BMI control or some were indirect neurons. Was this variability due to recording instability, session-to-session variability, or intentional changes in unit selection? Did it decrease substantially over the sessions? It would be useful to provide a graph of the number of units recorded each day from each NHP.

We thank the reviewer for pointing out the need for clarification. All units reported in the manuscript were actively used as control units in the BMI decoder; no indirect or non-control neurons were included. The variability in the number of recorded units across days reflects natural recording stability fluctuations associated with chronic microelectrode arrays rather than intentional changes in unit selection. As is typical with long-term implants, we observed a gradual downward trend in the total number of isolatable units over months, consistent with expected tissue responses around the array. However, the variation did not reflect substantial day-to-day drops within individual sessions. We have now revised the Methods section to clarify that (1) all units were used for BMI control, (2) variability was driven by chronic recording stability rather than experimental choices, and (3) a decline occurred over the implant duration.

If the neurons controlling the BMI change day to day substantially, did it affect performance? Is there a correlation between the number of neurons in the ensemble and learning?

We appreciate the reviewer’s question regarding the relationship between ensemble size and behavioral performance. In our dataset, session-to-session variability in the number of available units did not measurably affect BMI performance. This is expected because the Kalman filter used in this study requires only a small number of well-modulated units to provide stable two-dimensional velocity estimates; in principle, two directionally informative neurons are sufficient for cursor control. Across sessions, both monkeys demonstrated consistent baseline performance despite natural fluctuations in unit count, indicating that the decoder operated well within a regime where the number of neurons was more than adequate for robust control. Consistent with this, we did not observe a significant correlation between ensemble size and behavioral performance or learning metrics. We have added clarifying text in the manuscript to make this explicit.

The description “the subject performs a 10-minute center-out BMI task with gradually decaying computer assistance” is too vague and omits critical methodological details. The paper does not specify the type of assistance provided or how it was implemented. Was this a standard closed-loop decoder adaptation (CLDA) approach, or a different method? Without this information, it is impossible to interpret how the animals transitioned from assisted to independent control. The authors must clearly describe the algorithm or procedure used, including how assistance decayed over time and how this affected cursor dynamics. This information is essential for reproducing the experiment and understanding the learning process.

We thank the reviewer for highlighting the need for a more detailed description of the decoder-assistance procedure. We have now substantially expanded this section of the Methods to clarify that we implemented a standard closed-loop decoder adaptation (CLDA) procedure, following prior work by Orsborn et al. (2014) and Dangi et al. (2014). Specifically, we used a recursive maximum likelihood adaptation rule during a brief 5–10 minute calibration period prior to the main task. During CLDA, the cursor velocity was driven by a weighted combination of the neural decoder output and straight-line computer assistance. The assistance weight decreased linearly from 10% to 0% across the adaptation period, allowing the decoder to converge toward an intuitive mapping while the animal gradually assumed full control. Importantly, no CLDA or computer assistance was applied during the main baseline or perturbation blocks. These revisions clarify how the subjects transitioned from assisted to fully independent control and provide sufficient detail for experimental reproduction. The full procedural description is now included in the revised Methods section.

The distinction between easy and hard trials is introduced only in the results, leaving key aspects of experimental design unclear. The manuscript lacks definitions of each condition, their sequence, whether the order was randomized, and the total number of trials. Without this information, it is difficult to evaluate the task or interpret the findings. The authors should include a complete task description in the methods.

We thank the reviewer for noting the need for clearer definitions of the easy and hard task conditions. We have now revised the Methods section to explicitly describe the perturbation magnitudes, session structure, and trial counts. Each recording session consisted of one perturbation condition only: either the easy condition (50° rotation) or the hard condition (90° rotation). Sessions were not intermixed; all easy-condition sessions were completed before the hard condition sessions. For Monkey A, we recorded 16 easy and 18 hard sessions; for Monkey B, 17 easy and 24 hard sessions. Within each session, monkeys performed 336 trials in the baseline block and 384 trials in the perturbation block. These details have now been added to the Methods section to ensure clarity and reproducibility.

2- Classifying neuronal populations using only one parameter, spike waveform width, is an accepted but limited method. While this method appears in several NHP studies, rodent research typically uses multiple features (e.g., waveform, firing rate, autocorrelograms) to ensure more reliable separation of inhibitory and excitatory neurons. Although single-metric classification may be standard in some NHP work, this limitation should at least be acknowledged in the discussion, given the central focus on cell-type-specific contributions.

We thank the reviewer for this insightful comment. We agree that classifying neurons based solely on waveform width is a useful but limited approach, and that multispectral clustering using additional features (e.g., firing rate, autocorrelogram structure) is more common in rodent electrophysiology. In nonhuman primate recordings, however, single-metric spike-width classification is widely used and has been shown to reliably separate putative inhibitory and excitatory neurons. Our classification yielded NW proportions (27.2% in Monkey A; 19.3% in Monkey B) that fall well within the expected 15–30% range reported in prior NHP studies. Nevertheless, we have now added explicit acknowledgment of this methodological limitation in the Discussion and clarified that future work incorporating multi-feature classification or optogenetic tagging would further strengthen neuron-type identification. The revised text appears in the Discussion section.

3- Even accepting the use of a single waveform metric, the authors’ implementation raises concerns. The data clearly shows a bimodal distribution with reasonable Gaussian fits, yet the chosen cutoff excludes nearly half of the NW distribution while retaining most of the BW population. This asymmetry is difficult to justify. The studies used as references are not consistent with the cutoff, so this choice is neither standard nor well-motivated. Even more since they do seem to use the full Gaussian fit (e.g. Ardid et al. 2015 Fig.1D, Oemisch et al. 2015 Fig. 6C, Oemisch et al. 2019 Fig. 8B-G) Selectively trimming one population risks biasing the analysis and likely biases the role of NW neurons in adaptation. If the authors wish to maintain this threshold, they must justify why this specific cutoff is preferable to other options, such as using the full Gaussian fits, and provide a supplemental analysis showing how the results change with more symmetrical thresholds. This is critical to assess whether the conclusions depend on the current classification scheme.

We thank the reviewer for raising this important point. Our classification procedure follows the same likelihood-ratio approach used in Ceccarelli et al. (2023) and other primate electrophysiology studies. In this method, a two-Gaussian mixture model is fit to the trough-to-peak interval distribution, and neurons are assigned to the NW or BW class only when the probability of belonging to that class is at least 10× higher than the probability of belonging to the other class. This strategy provides a conservative and principled way to classify neurons by retaining only those with high assignment certainty.

The asymmetry noted by the reviewer arises naturally from the different variances of the NW and BW waveform distributions. The NW waveform distribution is considerably narrower, which shifts the 10× likelihood boundary closer to its peak. This does not reflect selective removal of NW neurons but rather the statistical structure of the underlying data, which is the same reason similar asymmetry appears in the published literature using this method. Because our goal was to compare neuron groups with high classification confidence, we chose the likelihood-ratio approach instead of the Gaussian-intersection method, which assigns a larger fraction of borderline neurons whose waveform properties overlap. We have now clarified this rationale in the manuscript and added a brief acknowledgment in the Discussion noting that, while alternative thresholds exist, our conclusions rely on the strongly classified subset of neurons and therefore remain robust to the conservative nature of the cutoff.

4- Several statements in the manuscript conflate BMI learning with natural motor learning without sufficient support or discussion. While it is very likely that BMI adaptation recruits the same mechanisms, potentially by hijacking the same cortico-striatal-thalamo-cortical loops involved in motor learning, there is also clear evidence of differences. Prior work, including Zippi et al. (2023) (https://doi.org/10.1038/s41598-023-44405-y), has shown distinct neural representations during BMI versus manual control across the motor cortex, prefrontal cortex, and striatum. These differences may arise from the number and identity of neurons recruited in each case or because they do use different systems altogether. The authors should acknowledge this nuance, avoid treating BMI learning as equivalent to motor learning, and discuss these distinctions explicitly.

We thank the reviewer for this insightful comment. We agree that BMI-based adaptation and natural motor learning share overlapping computational principles but are not equivalent processes. To prevent confusion, we have revised the manuscript to refer to the phenomenon under study as “neurofeedback adaptation” rather than “motor learning,” except when discussing prior literature. We also added text in the Introduction and Discussion explicitly noting that BMI adaptation occurs within an artificial neural-to-cursor mapping and lacks the proprioceptive and musculoskeletal feedback present in natural movement, consistent with evidence from Zippi et al. (2023) and others showing distinct neural representations between BMI and manual control. These revisions clarify that our findings pertain specifically to neurofeedback adaptation within a BMI framework and are not intended to imply direct equivalence with natural motor learning.

5- Figure 2C shows a clear and important result: NW neurons decrease their firing rate from easy to hard conditions, while BW neurons increase theirs. This finding directly contradicts the overall narrative of the paper, which emphasizes a dominant role for NW neurons in adaptation. Despite this, the authors barely mention the result and provide no interpretation. Is it possible that the increased firing rate in BW neurons reflects a compensatory response to the harder perturbation? If so, how does this fit with the conclusion that NW neurons play the dominant role in adaptation? This apparent contradiction needs to be addressed directly.

We thank the reviewer for raising this important point. We agree that the firing-rate differences observed between the easy and hard conditions require clarification. Because our recordings were obtained across different sessions, the neurons contributing to the easy-condition dataset are not the same neurons contributing to the hard-condition dataset. Therefore, cross-session comparisons of mean firing rate cannot be interpreted as learning-related increases or decreases in activity. These differences instead reflect normal variability in the set of isolatable units recorded each day.

Our intention in presenting Figure 2C was not to use firing-rate changes across conditions to infer adaptation-related effects, but rather to illustrate the intrinsic firing-rate differences between waveform classes. Based on waveform shape, NW neurons are more likely to correspond to interneurons, whereas BW neurons are more likely to correspond to pyramidal cells, and the observed firing-rate pattern is consistent with these general physiological trends reported in the literature. This interpretation relates to intrinsic cell-type properties rather than task-dependent modulation.

Crucially, all conclusions about the dominant role of NW neurons in adaptation are based on within-session comparisons, such as unit contribution, pSOT coordination, and ω² encoding, where the same neurons are compared across baseline and perturbation blocks. These within-session metrics directly capture how each neuronal phenotype responds to the perturbation and therefore form the proper basis for our interpretation. We have revised the Results and Discussion to clarify this distinction.

6- Is there a difference in ω2 between early and late adaptation, or between baseline and late adaptation? How much does the explained var

Attachment Submitted filename: Response to reviewers.pdf

# Peer review: Decision Letter 1

**DOI:** 10.1371/journal.pone.0351053.r004

**Authors:** Marie-Constance Corsi, Marie-Constance Corsi

17 Mar 2026

PONE-D-25-29925R1

Distinct roles of neuronal phenotypes during neurofeedback adaptation

PLOS One

Dear Dr. Santacruz,

Thank you for submitting your manuscript to PLOS ONE. After careful consideration, we feel that it has merit but does not fully meet PLOS ONE’s publication criteria as it currently stands. Therefore, we invite you to submit a revised version of the manuscript that addresses the points raised during the review process.

The authors have addressed most of the previous reviewer's comments and improved the manuscript's clarity. However, two substantive concerns remain before the manuscript can be considered for acceptance

Please submit your revised manuscript by May 01 2026 11:59PM. If you will need more time than this to complete your revisions, please reply to this message or contact the journal office at plosone@plos.org. When you're ready to submit your revision, log on to https://www.editorialmanager.com/pone/ and select the 'Submissions Needing Revision' folder to locate your manuscript file.

Please include the following items when submitting your revised manuscript:

- A letter that responds to each point raised by the academic editor and reviewer(s). You should upload this letter as a separate file labeled 'Response to Reviewers'.
- A marked-up copy of your manuscript that highlights changes made to the original version. You should upload this as a separate file labeled 'Revised Manuscript with Track Changes'.
- An unmarked version of your revised paper without tracked changes. You should upload this as a separate file labeled 'Manuscript'.

-->If you would like to make changes to your financial disclosure, please include your updated statement in your cover letter. Guidelines for resubmitting your figure files are available below the reviewer comments at the end of this letter.

If applicable, we recommend that you deposit your laboratory protocols in protocols.io to enhance the reproducibility of your results. Protocols.io assigns your protocol its own identifier (DOI) so that it can be cited independently in the future. For instructions see: https://journals.plos.org/plosone/s/submission-guidelines#loc-laboratory-protocols. Additionally, PLOS ONE offers an option for publishing peer-reviewed Lab Protocol articles, which describe protocols hosted on protocols.io. Read more information on sharing protocols at https://plos.org/protocols?utm_medium=editorial-email&utm_source=authorletters&utm_campaign=protocols.

We look forward to receiving your revised manuscript.

Kind regards,

Marie-Constance Corsi

Academic Editor

PLOS One

**Journal Requirements:**

1. If the reviewer comments include a recommendation to cite specific previously published works, please review and evaluate these publications to determine whether they are relevant and should be cited. There is no requirement to cite these works unless the editor has indicated otherwise.

2. Please review your reference list to ensure that it is complete and correct. If you have cited papers that have been retracted, please include the rationale for doing so in the manuscript text, or remove these references and replace them with relevant current references. Any changes to the reference list should be mentioned in the rebuttal letter that accompanies your revised manuscript. If you need to cite a retracted article, indicate the article’s retracted status in the References list and also include a citation and full reference for the retraction notice.

[Note: HTML markup is below. Please do not edit.]

Reviewers' comments:

Reviewer's Responses to Questions

**Comments to the Author**

1. If the authors have adequately addressed your comments raised in a previous round of review and you feel that this manuscript is now acceptable for publication, you may indicate that here to bypass the “Comments to the Author” section, enter your conflict of interest statement in the “Confidential to Editor” section, and submit your "Accept" recommendation.

Reviewer #1: (No Response)

**********

2. Is the manuscript technically sound, and do the data support the conclusions?

The manuscript must describe a technically sound piece of scientific research with data that supports the conclusions. Experiments must have been conducted rigorously, with appropriate controls, replication, and sample sizes. The conclusions must be drawn appropriately based on the data presented.

Reviewer #1: Yes

**********

3. Has the statistical analysis been performed appropriately and rigorously?

Reviewer #1: Yes

**********

4. Have the authors made all data underlying the findings in their manuscript fully available?

The PLOS Data policy requires authors to make all data underlying the findings described in their manuscript fully available without restriction, with rare exception (please refer to the Data Availability Statement in the manuscript PDF file). The data should be provided as part of the manuscript or its supporting information, or deposited to a public repository. For example, in addition to summary statistics, the data points behind means, medians and variance measures should be available. If there are restrictions on publicly sharing data—e.g. participant privacy or use of data from a third party—those must be specified.

Reviewer #1: No

**********

5. Is the manuscript presented in an intelligible fashion and written in standard English?

PLOS ONE does not copyedit accepted manuscripts, so the language in submitted articles must be clear, correct, and unambiguous. Any typographical or grammatical errors should be corrected at revision, so please note any specific errors here.

Reviewer #1: Yes

**********

6. Review Comments to the Author

Please use the space provided to explain your answers to the questions above. You may also include additional comments for the author, including concerns about dual publication, research ethics, or publication ethics. (Please upload your review as an attachment if it exceeds 20,000 characters)

Reviewer #1: Authors addressed most of the previous review and improved the clarity of the manuscript. However, two concerns remain:

1- Figure 2A. Unfortunately, I don't believe the authors adequately addressed previous concerns about the cutoff in the neuron selection. I understand that previous studies have used a 1:10 likelihood criterion as a conservative way to define high-confidence classes (Ceccarelli et al. (2023) ). However, in the present dataset (with one Gaussian centered at ~0.25 and the other at ~0.47), the resulting cutoffs appear shifted (0.25 and 0.35)and split (at 0.25) of one of the Gaussian distributions in the middle rather than lying in the overlap region between the two fitted populations (as happens in Ceccarelli et al. (2023). This selection may reduce statistical power and bias the analysis. Moreover, many neurons are excluded and then the analysis may no longer represent the underlying population and may be very dependent on the chosen cutoff.

Is it possible that the histogram is not showing the entire dataset? (so the Gaussian obtained is not centered at 0.25?). Is it possible that the histogram shows the mean trough-to-peak interval while the Gaussian was fit to the raw data? The authors should consider showing the Gaussian fits overlaid on the histogram to assess any misalignment. However, if the Gaussian was fit correctly and the cutoff lies within one Gaussian, the authors should show how the cutoff changes under less stringent likelihood ratios (e.g., 1:5 or 1:3) and confirm that the main result remain unchanged across at least one of these thresholds.

2- Figure 2C. I understand that firing-rate differences between these conditions cannot be interpreted as within-neuron learning effects. However, Figure 2C still shows a clear population-level pattern. NW neurons have lower firing rates in the hard condition than in the easy condition. The converse is true for BW neurons. Even if this reflects differences in the neurons recorded across sessions, the pattern remains in the data and should be addressed. The cross-session design also does not prevent statistical comparison of the populations. A simple independent-samples test could resolve this concern. If the effect were significant, it could indicate a shift in the types of neurons engaged across different task conditions.

(minor) Data was still not accessible through the github link provided by the authors.

**********

7. PLOS authors have the option to publish the peer review history of their article (what does this mean?). If published, this will include your full peer review and any attached files.

If you choose “no”, your identity will remain anonymous but your review may still be made public.

**Do you want your identity to be public for this peer review?**  For information about this choice, including consent withdrawal, please see our Privacy Policy.

Reviewer #1: **Yes:**  Nuria Vendrell Llopis

**********

[NOTE: If reviewer comments were submitted as an attachment file, they will be attached to this email and accessible via the submission site. Please log into your account, locate the manuscript record, and check for the action link "View Attachments". If this link does not appear, there are no attachment files.]

To ensure your figures meet our technical requirements, please review our figure guidelines: https://journals.plos.org/plosone/s/figures

You may also use PLOS’s free figure tool, NAAS, to help you prepare publication quality figures: https://journals.plos.org/plosone/s/figures#loc-tools-for-figure-preparation.

NAAS will assess whether your figures meet our technical requirements by comparing each figure against our figure specifications.

# Author comment: Author response to Decision Letter 2

**DOI:** 10.1371/journal.pone.0351053.r005

5 May 2026

REVIEWER #1:

1- Figure 2A. Unfortunately, I don't believe the authors adequately addressed previous concerns about the cutoff in the neuron selection. I understand that previous studies have used a 1:10 likelihood criterion as a conservative way to define high-confidence classes (Ceccarelli et al. (2023) ). However, in the present dataset (with one Gaussian centered at ~0.25 and the other at ~0.47), the resulting cutoffs appear shifted (0.25 and 0.35) and split (at 0.25) of one of the Gaussian distributions in the middle rather than lying in the overlap region between the two fitted populations (as happens in Ceccarelli et al. (2023). This selection may reduce statistical power and bias the analysis. Moreover, many neurons are excluded and then the analysis may no longer represent the underlying population and may be very dependent on the chosen cutoff.

Is it possible that the histogram is not showing the entire dataset? (so the Gaussian obtained is not centered at 0.25?). Is it possible that the histogram shows the mean trough-to-peak interval while the Gaussian was fit to the raw data? The authors should consider showing the Gaussian fits overlaid on the histogram to assess any misalignment. However, if the Gaussian was fit correctly and the cutoff lies within one Gaussian, the authors should show how the cutoff changes under less stringent likelihood ratios (e.g., 1:5 or 1:3) and confirm that the main result remain unchanged across at least one of these thresholds.

We appreciate the reviewer’s careful attention to detail and we have taken the suggestion onboard. Firstly, we would like to confirm that the entire dataset is shown in the histogram in Fig. 2A. Secondly, following the suggestion to assess the stability of our findings under less stringent likelihood ratios, we have re-analyzed the entire dataset using a 1:5 likelihood ratio. As expected, this shifted the NW and BW cutoffs further in the right and left tails of the distributions, respectively.

NW Threshold: New (Old) BW Threshold: New (Old)

Monkey A 0.275 ms (0.243 ms) 0.348 ms (0.360 ms)

Monkey B 0.272 ms (0.240 ms) 0.360 ms (0.373 ms)

Regarding the impact of the threshold shift, we found that by relaxing the threshold, we significantly increased the number of neurons included in the analysis, thereby addressing the concern regarding the potential exclusion of representative population data.

# NW Neurons: New (Old) # BW: New (Old) Total # Neurons Recorded

Monkey A 430 (325) 537 (513) 1195

Monkey B 933 (737) 2107 (2146*) 3814

*We would like to note that we previously noted that 2,146 neurons were labeled as BW; however, the correct count under the 1:10 ratio was 2,046. By adopting the 1:5 likelihood ratio and adjusting the BW threshold for Monkey B (from 0.373 ms to 0.360 ms), the number of BW neurons did increase.

With the neuron classification performed this way, we found that the primary conclusions of the study remained robust under the 1:5 criterion. All previously reported effects were preserved using the 1:5 ratio, with one exception. We updated Figures 2, 3, 4, 5, and 6, and Supplementary Figures 1 and 3, to reflect visualization of the re-analyzed data. The corresponding statistical analyses were also updated accordingly and incorporated into the revised manuscript. The singular result for which statistical significance was not preserved was the main effect of neuron type on the peak percentage of explained variance (peak ω^2; see Figure 6) in one of the subjects. Specifically, two-way ANOVA with peak ω^2 as the dependent variable found that the main effect of neuron type had a p-value of p = 0.052 for monkey A (the main effect is significant for monkey b is still significant: p << 0.001), which is slightly above the conventional significance level of 0.05. We note in the revised text that the effect is trending for monkey A and significant for monkey B.

2- Figure 2C. I understand that firing-rate differences between these conditions cannot be interpreted as within-neuron learning effects. However, Figure 2C still shows a clear population-level pattern. NW neurons have lower firing rates in the hard condition than in the easy condition. The converse is true for BW neurons. Even if this reflects differences in the neurons recorded across sessions, the pattern remains in the data and should be addressed. The cross-session design also does not prevent statistical comparison of the populations. A simple independent-samples test could resolve this concern. If the effect were significant, it could indicate a shift in the types of neurons engaged across different task conditions.

We agree that we should have set up our original analysis to capture this in the way that you suggest. To better comprehensively reveal what effects are truly present in the data depicted in Fig 2C, we have performed a two-way ANOVA that simultaneously tests for main effects between task conditions and neuron types, as suggested.

TWO-WAY ANOVA p-value (Monkey A) p-value (Monkey B)

Main effect: task condition p = 0.637 p = 0.773

Main effect: neuron type p = 0.000006 p = 8.15 × 10⁻63

Interaction p = 0.0026 p = 0.348

Our results confirm a significant effect of neuron type, as originally reported in the paper, and this result holds for both subjects (see summary table below for your convenience). While the data presented in Fig 2C does visually suggest there is may be a main effect of task condition, we found that task condition was not a significant main effect and that this also held true for both subjects. Although a significant interaction was detected in one subject (monkey A), the absence of a main effect of task condition and the lack of a reproducible interaction across both subjects suggests that these trends likely reflect session-specific variance rather than a fundamental shift in population engagement. We have added these statistical details to the manuscript to clarify that while neuron type is a robust predictor of firing rate, task difficulty did not exert a consistent or significant influence on firing rates.

We have updated the following text in the Neuron Classification subsection within the Results:

We evaluated how firing rate activity of neurons differ under this classification and across task conditions. The absence of an effect of task condition (Monkey A: p = 0.637; Monkey B: p = 0.773; main effect of neuron type, two-way ANOVA) and the lack of a reproducible interaction between task condition and neuron type across both subjects (Monkey A: p = 0.0026; Monkey B: p = 0.348; interaction effect, two-way ANOVA) suggest that the primary driver of differences in firing rate is the neuron type (Monkey A: p <0.001; Monkey B: p<0.001; main effect of neuron type, two-way ANOVA). The mean firing rate of NW neurons during cursor movements was significantly higher than that of BW neurons (Fig. 2C) under both easy and hard task conditions. This high firing rate of NW neurons, which is typical of inhibitory neuron, is also consistent with findings from previous studies[49,58,60].

3 (minor) Data was still not accessible through the github link provided by the authors.

We recently changed how our data is hosted so that it is no longer linked to individual trainee Github accounts. Access to our data is restricted now while under review, but if accepted the data will be freely available on figshare (this platform allows for larger repositories). We will comply with the journal’s requirements to make the data available upon publication but understand from their policy that it is permissible to maintain the data privately during the manuscript review process.

Attachment Submitted filename: Response_to_Reviewers_auresp_2.pdf

# Peer review: Decision Letter 2

**DOI:** 10.1371/journal.pone.0351053.r006

**Authors:** Marie-Constance Corsi, Marie-Constance Corsi, Marie-Constance Corsi

21 May 2026

<p>Distinct roles of neuronal phenotypes during neurofeedback adaptation

PONE-D-25-29925R2

Dear Dr. Santacruz,

We’re pleased to inform you that your manuscript has been judged scientifically suitable for publication and will be formally accepted for publication once it meets all outstanding technical requirements.

Within one week, you’ll receive an e-mail detailing the required amendments. When these have been addressed, you’ll receive a formal acceptance letter and your manuscript will be scheduled for publication.

An invoice will be generated when your article is formally accepted. Please note, if your institution has a publishing partnership with PLOS and your article meets the relevant criteria, all or part of your publication costs will be covered. Please make sure your user information is up-to-date by logging into Editorial Manager at Editorial Manager® and clicking the ‘Update My Information' link at the top of the page. For questions related to billing, please contact billing support.

If your institution or institutions have a press office, please notify them about your upcoming paper to help maximize its impact. If they’ll be preparing press materials, please inform our press team as soon as possible -- no later than 48 hours after receiving the formal acceptance. Your manuscript will remain under strict press embargo until 2 pm Eastern Time on the date of publication. For more information, please contact onepress@plos.org.

Kind regards,

Marie-Constance Corsi

Academic Editor

PLOS One

Additional Editor Comments (optional):

Reviewers' comments:

Reviewer's Responses to Questions

**Comments to the Author**

1. If the authors have adequately addressed your comments raised in a previous round of review and you feel that this manuscript is now acceptable for publication, you may indicate that here to bypass the “Comments to the Author” section, enter your conflict of interest statement in the “Confidential to Editor” section, and submit your "Accept" recommendation.

Reviewer #1: All comments have been addressed

**********

2. Is the manuscript technically sound, and do the data support the conclusions?

The manuscript must describe a technically sound piece of scientific research with data that supports the conclusions. Experiments must have been conducted rigorously, with appropriate controls, replication, and sample sizes. The conclusions must be drawn appropriately based on the data presented.

Reviewer #1: Yes

**********

3. Has the statistical analysis been performed appropriately and rigorously?

Reviewer #1: Yes

**********

4. Have the authors made all data underlying the findings in their manuscript fully available?

The PLOS Data policy requires authors to make all data underlying the findings described in their manuscript fully available without restriction, with rare exception (please refer to the Data Availability Statement in the manuscript PDF file). The data should be provided as part of the manuscript or its supporting information, or deposited to a public repository. For example, in addition to summary statistics, the data points behind means, medians and variance measures should be available. If there are restrictions on publicly sharing data—e.g. participant privacy or use of data from a third party—those must be specified.

Reviewer #1: Yes

**********

5. Is the manuscript presented in an intelligible fashion and written in standard English?

PLOS ONE does not copyedit accepted manuscripts, so the language in submitted articles must be clear, correct, and unambiguous. Any typographical or grammatical errors should be corrected at revision, so please note any specific errors here.

Reviewer #1: Yes

**********

6. Review Comments to the Author

Please use the space provided to explain your answers to the questions above. You may also include additional comments for the author, including concerns about dual publication, research ethics, or publication ethics. (Please upload your review as an attachment if it exceeds 20,000 characters)

Reviewer #1: (No Response)

**********

7. PLOS authors have the option to publish the peer review history of their article (what does this mean?). If published, this will include your full peer review and any attached files.

If you choose “no”, your identity will remain anonymous but your review may still be made public.

**Do you want your identity to be public for this peer review?**  For information about this choice, including consent withdrawal, please see our Privacy Policy.

Reviewer #1: **Yes:**  Nuria Vendrell Llopis

**********

# Editor report: Acceptance letter

**DOI:** 10.1371/journal.pone.0351053.r007

**Authors:** Marie-Constance Corsi, Marie-Constance Corsi, Marie-Constance Corsi

PONE-D-25-29925R2

PLOS One

Dear Dr. Santacruz,

I'm pleased to inform you that your manuscript has been deemed suitable for publication in PLOS One. Congratulations! Your manuscript is now being handed over to our production team.

At this stage, our production department will prepare your paper for publication. This includes ensuring the following:

* All references, tables, and figures are properly cited

* All relevant supporting information is included in the manuscript submission,

* There are no issues that prevent the paper from being properly typeset

You will receive further instructions from the production team, including instructions on how to review your proof when it is ready. Please keep in mind that we are working through a large volume of accepted articles, so please give us a few days to review your paper and let you know the next and final steps.

Lastly, if your institution or institutions have a press office, please let them know about your upcoming paper now to help maximize its impact. If they'll be preparing press materials, please inform our press team within the next 48 hours. Your manuscript will remain under strict press embargo until 2 pm Eastern Time on the date of publication. For more information, please contact onepress@plos.org.

You will receive an invoice from PLOS for your publication fee after your manuscript has reached the completed accept phase. If you receive an email requesting payment before acceptance or for any other service, this may be a phishing scheme. Learn how to identify phishing emails and protect your accounts at https://explore.plos.org/phishing.

If we can help with anything else, please email us at customercare@plos.org.

Thank you for submitting your work to PLOS ONE and supporting open access.

Kind regards,

PLOS ONE Editorial Office Staff

on behalf of

Dr. Marie-Constance Corsi

Academic Editor

PLOS One
